# Advancing atomic electron tomography with neural networks

**DOI:** 10.1186/s42649-025-00113-7

**Published:** 2025-06-19

**Authors:** Juhyeok Lee, Yongsoo Yang

**Affiliations:** 1 Energy Geosciences Division, Lawrence Berkeley National Laboratory, Berkeley, CA 94720 USA; 2 National Center for Electron Microscopy, Lawrence Berkeley National Laboratory, Berkeley, CA 94720 USA; 3 Department of Physics, Korea Advanced Institute of Science and Technology (KAIST), Daejeon, 34141 Republic of Korea; 4 Graduate School of Semiconductor Technology, School of Electrical Engineering, Korea Advanced Institute of Science and Technology (KAIST), Daejeon, 34141 Republic of Korea

**Keywords:** Atomic electron tomography, Neural network, 3D structural analysis

## Abstract

Accurate determination of three-dimensional (3D) atomic structures is crucial for understanding and controlling the properties of nanomaterials. Atomic electron tomography (AET) offers non-destructive atomic imaging with picometer-level precision, enabling the resolution of defects, interfaces, and strain fields in 3D, as well as the observation of dynamic structural evolution. However, reconstruction artifacts arising from geometric limitations and electron dose constraints can hinder reliable atomic structure determination. Recent progress has integrated deep learning, especially convolutional neural networks, into AET workflows to improve reconstruction fidelity. This review highlights recent advances in neural network-assisted AET, emphasizing its role in overcoming persistent challenges in 3D atomic imaging. By significantly enhancing the accuracy of both surface and bulk structural characterization, these methods are advancing the frontiers of nanoscience and enabling new opportunities in materials research and technology.

**Supplementary Information:**

The online version contains supplementary material available at 10.1186/s42649-025-00113-7.

## Introduction

In recent years, the demand for innovative nanomaterials and nanostructures has surged across diverse fields, including catalysis [1–9], electronics [10–15], energy storage [16–22], quantum technologies [23–28], structural materials [29–34], biosensing [27, 35–38], and targeted drug delivery [39–42]. Designing materials with tailored functionalities requires precise control over the arrangement of atoms—the fundamental building blocks of matter [43–46]. While exceptions exist, such as atomic chains and purely two-dimensional (2D) materials like monolayer graphene, most materials, even those classified as 0-dimensional, 1-dimensional, or 2D, possess inherently three-dimensional (3D) atomic arrangements. To fully manipulate these structures, it is essential to not only measure atomic positions with high precision but also understand how they evolve under external influences such as temperature, electromagnetic fields, and pressure.

For decades, transmission electron microscopy (TEM) has been a primary tool for atomic-scale structural analysis; more recently, the advent of aberration correctors has significantly enhanced achievable resolution, making atomic resolution imaging routinely attainable [47–54]. However, TEM images provide only 2D projections of 3D structures, limiting their ability to capture atomic arrangements in all three dimensions [55–60]. Scanning probe techniques can resolve surface features at the atomic scale but lack access to subsurface structures [61–66]. Crystallographic methods using X-rays, electrons, or neutrons enable high-resolution 3D structural determination [67–73] but are largely restricted to periodic crystals, making them unsuitable for studying non-crystalline features such as grain boundaries, dislocations, interfaces, and point defects [69, 70, 73, 74]. Other techniques, like coherent diffractive imaging, have shown promise for 2D and 3D analysis but have not yet achieved true atomic resolution [75–81]. While atom probe tomography can provide 3D atomic information, it is a destructive technique and is challenging to apply for dynamic studies [82–86].

**Fig. 1:**
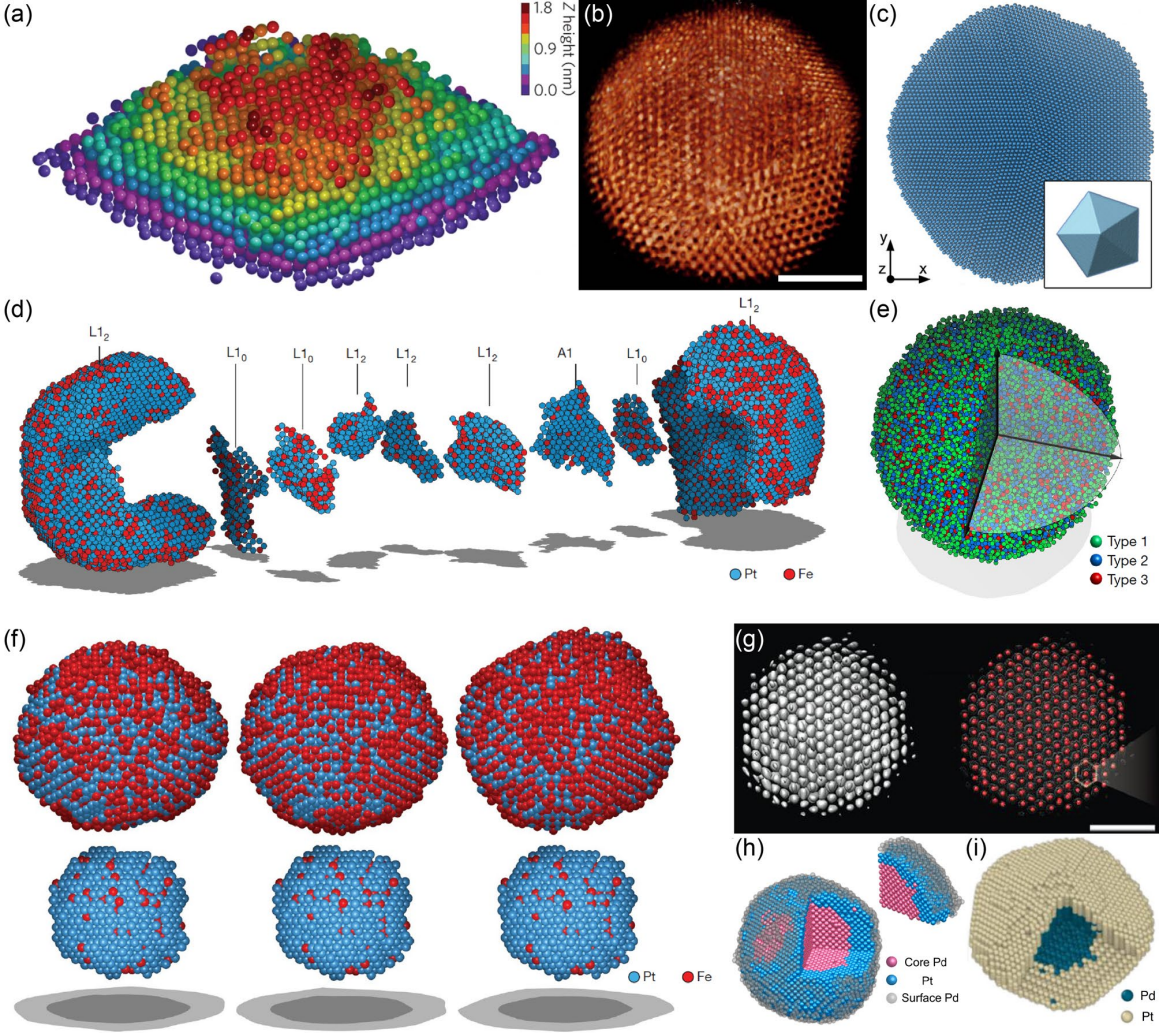
Representative 3D atomic structural analysis based on electron tomography. (**a**) 3D positions of individual atoms in a tungsten needle sample. The 3D atomic structure consists of nine atomic layers along the [011] direction, labelled with dark red, red, orange, yellow, green, cyan, blue, magenta and purple from layers 1–9, respectively. Adapted from Reference [93], © 2015 Springer Nature. (**b**) A three-dimensional reconstruction of a Ag-Au nanocluster, showing atomic structure and composition of the cluster. Scale bar, 2 nm. Adapted from Reference [92], © 2015 Macmillan Publishers Limited. (**c**) 3D visualization of a reconstructed Au nanodecahedron containing more than 90 000 atoms. Adapted from Reference [91], this is an unofficial adaptation of an article that appeared in an ACS publication. ACS has not endorsed the content of this adaptation or the context of its use. © 2015 American Chemical Society. (**d**) Experimentally determined complex grain structure of an FePt nanoparticle via atomic electron tomography. Adapted from Reference [95], © 2017 Springer Nature. (**e**) Experimental 3D atomic model of an amorphous nanoparticle composed of eight chemical elements. Adapted from Reference [106] with permission, © 2021 Springer Nature. (**f**) 3D atomic models of an FePt nanoparticle after 9 min (left), 16 min (middle), and 26 min (right) of accumulated annealing. The top row shows the entire nanoparticle, while the bottom row highlights the Pt-rich core at each stage. Adapted from Reference [111], © 2019 Springer Nature. (**g**) 3D density maps and atomic positions of a single-crystalline Pt nanocrystal along the [111] zone axis. Scale bar, 1 nm. Adapted from Reference [110] with permission, © 2020 AAAS. (**h-i**) Experimentally determined 3D atomic structures of Pd@Pt core-shell nanoparticles, revealing (**h**) strain correlation between the surface and interface and (**i**) chemical diffusion at the interface. Adapted from References [103, 104]

Electron tomography has emerged as a powerful tool for non-destructive 3D atomic structural analysis [87–89]. In 2012, it achieved near-atomic resolution (2.4 Å) without relying on crystalline symmetry [90]. In 2015, atomic electron tomography (AET) was demonstrated [91–93], achieving an atomic-level precision of 19 picometers by combining aberration-corrected TEM with advanced iterative reconstruction algorithms [93, 94]. This milestone enabled the direct determination of 3D atomic positions of individual atoms in nanomaterials, shifting AET from qualitative visualization to precise, quantitative materials characterization (Fig. 1a-c).

Since then, AET has been widely used to investigate complex non-crystalline atomic structures such as grain boundaries [95, 96], dislocations [97, 98], stacking faults [60, 90, 98], point defects [93, 95], local distortions [99–101], heterointerfaces [100, 102–105], amorphous structures [98, 106, 107], and strain tensors [60, 91, 93, 95, 103, 108–110] in unprecedented 3D detail (Fig. 1d-i). Leveraging its non-destructive nature, AET has also enabled 4D (3D space + time) atomic-resolution imaging, capturing dynamic structural changes over time, such as nucleation and growth processes [111–113]. Furthermore, experimentally determined atomic coordinates have been integrated into ab initio calculations, allowing direct correlations between atomic-scale structures and physical, chemical, and electronic properties of various materials [43, 90, 93, 95, 99, 108, 109, 114–116].

Despite its success, AET faces a major challenge stemming from geometric constraints in electron tomography experiments, where the specimen holder or grid obstructs the electron beam beyond certain tilt angles, preventing the full acquisition of angular data [58, 117]. This limitation, known as the “missing wedge” problem, introduces elongation along beam direction and Fourier ringing artifacts in reconstructed tomograms, leading to distortions that particularly affect surface atomic structures [117–119]. As a result, while AET has been widely applied to study internal atomic arrangements, achieving precise 3D surface atomic structure determination remains a persistent challenge.

This limitation is especially critical because surface atomic configurations govern key material properties and applications. For example, catalytic activity is almost entirely dictated by the arrangement of surface atoms, rather than the internal atomic structure [120]. Likewise, surface atomic structures influence adhesion [121], corrosion resistance [122], electronic transport [123, 124], and interfacial phenomena [125, 126] in a wide range of materials. Accurately determining the 3D atomic structure of surfaces is thus essential for both fundamental scientific insights and practical applications.

To address the missing wedge problem, various approaches have been explored, including deep learning-based neural networks. Convolutional neural networks (CNNs), in particular, have gained significant attraction in electron microscopy for tasks such as missing data reconstruction and super-resolution imaging [127–131]. Recent advancements have demonstrated the successful integration of AET with neural networks guided by the atomicity principle (which assumes that the sample consists solely of discrete atomic potentials), substantially improving the reliability of surface 3D atomic structural characterization [108, 132–135].

This review highlights recent advancements in AET, with a special focus on neural network-based methodologies. We discuss fundamental concepts behind neural network-assisted atomic-resolution tomography, explore experimental and computational success cases, and introduce novel approaches such as image inpainting for improving tomography tilt series before reconstruction. By leveraging machine learning, AET is poised to overcome long-standing limitations. This approach offers unprecedented precision in 3D atomic structure determination and paves the way for breakthroughs in materials science and nanotechnology.

### Deep learning for missing wedge recovery in AET

**Fig. 2:**
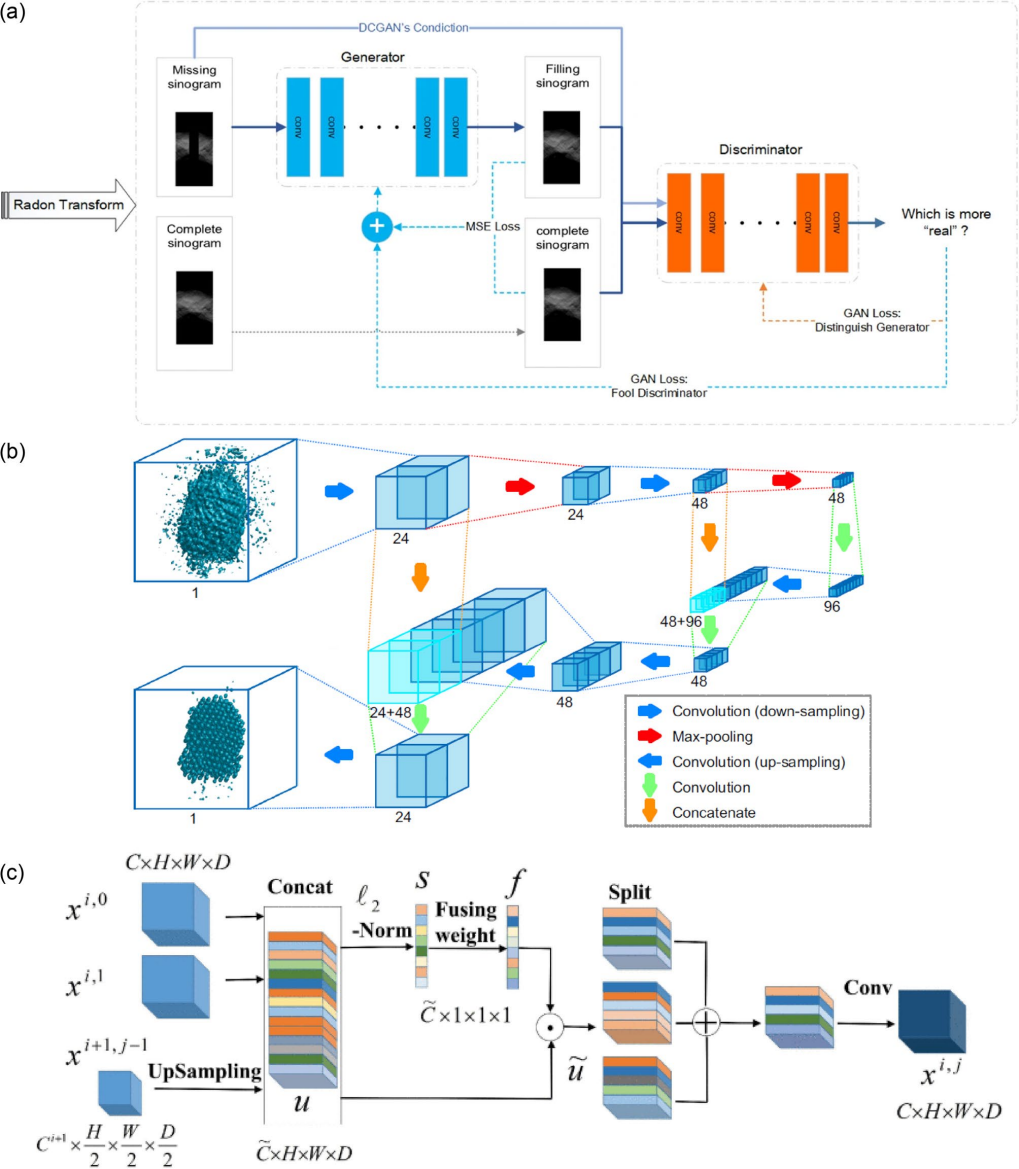
Neural network architectures for enhancing AET. (**a**) A residual-in-residual dense block-based GAN that fills missing regions in the sinogram domain as the first step of a two-stage process, followed by a U-Net-based network for artifact reduction in the reconstructed volume. Adapted from Reference [132]. (**b**) Architecture of the deep learning augmentation applied to 3D tomograms. The model follows a 3D U-Net structure, where each box represents a feature map. The number of channels is indicated below each feature map. Adapted from Reference [108]. (**c**) Overview of the skip fusing unit within the EC-UNETR framework. The outputs from the preceding subnetworks within the identical stage and the upsampled outputs from the lower stage are concatenated and normalized to calculate the fusing weights. Adapted from Reference [134] with permission, © 2024 IEEE

Recent advances have addressed the long-standing issue of missing wedge artifacts in AET by integrating machine learning to recover unmeasured information and enhance reconstruction quality [60, 108, 112, 131–135]. These approaches typically train neural networks to inpaint missing tilt projections or remove artifacts in reconstructed tomograms, thereby surpassing the limitations of conventional reconstruction algorithms. A notable example is the two-step deep learning pipeline developed by Ding et al., which first infills missing wedge data in the sinogram domain (a volume of collected tilt series) and subsequently refines the 3D reconstruction using a U-Net-based architecture [132]. The first step employs a generative adversarial network (GAN) to predict the data corresponding to missing tilt angles in the sinogram (Fig. 2a), while the second applies an encoder-decoder network with skip connections to suppress residual artifacts in the tomogram. This joint model produced reconstructions of significantly higher fidelity than traditional weighted back-projection [136] or simultaneous algebraic reconstruction technique [137] algorithms, even when over 80% of the tilt range was missing. The method was later extended to the information recovery and de-artifact model (IRDM), which achieved sub-angstrom resolution (0.7 Å) in the electron tomography of nanoporous gold [133]. These results convincingly demonstrate that deep learning can effectively mitigate the missing wedge problem in AET.

A further advancement was introduced in 2021, in which a 3D neural network was inserted as a post-reconstruction augmentation step in the AET reconstruction pipeline [108]. Lee et al. implemented a 3D U-Net architecture [108, 138] to enhance preliminary reconstructions obtained from iterative algorithms such as GENFIRE [139] (Fig. 2b). The initial “raw” tomograms often suffer from blurring and elongation due to the missing wedge. These tomograms were processed through the trained U-Net, resulting in refined volumes. In the output, atomic sites appear as well-isolated peaks with the expected Gaussian intensity profiles. The tomogram augmentation approach also introduced a unique and powerful prior, known as the atomicity constraint, which is specifically tailored for atomic electron tomography. This prior is based on the assumption that the sample consists of discrete atomic potentials. As a result, the network learned to transform blurred density distributions into well-resolved atomic peaks, even when applied to structures that were entirely different from those used during training. This approach led to a substantial increase in reconstruction accuracy, achieving an atomic coordinate precision of 15.1 pm.

More recently, Yu et al. introduced further improvements by developing an ensemble cross U-Net transformer model (EC-UNETR) [134] (Fig. 2c). The EC-UNETR design incorporates hierarchical subnetworks that process features along orthogonal axes and includes a skip fusing unit to improve network stability and flexibility. Transformer-based attention mechanisms are embedded within both the encoder and decoder stages, enabling the model to capture long-range spatial dependencies. This architecture improved the recovery of fine structural details, reduced reconstruction artifacts, and yielded a 5.5% decrease in root-mean-square error compared to earlier models.

### Precise determination of Pt nanoparticle surfaces and interface structures

**Fig. 3:**
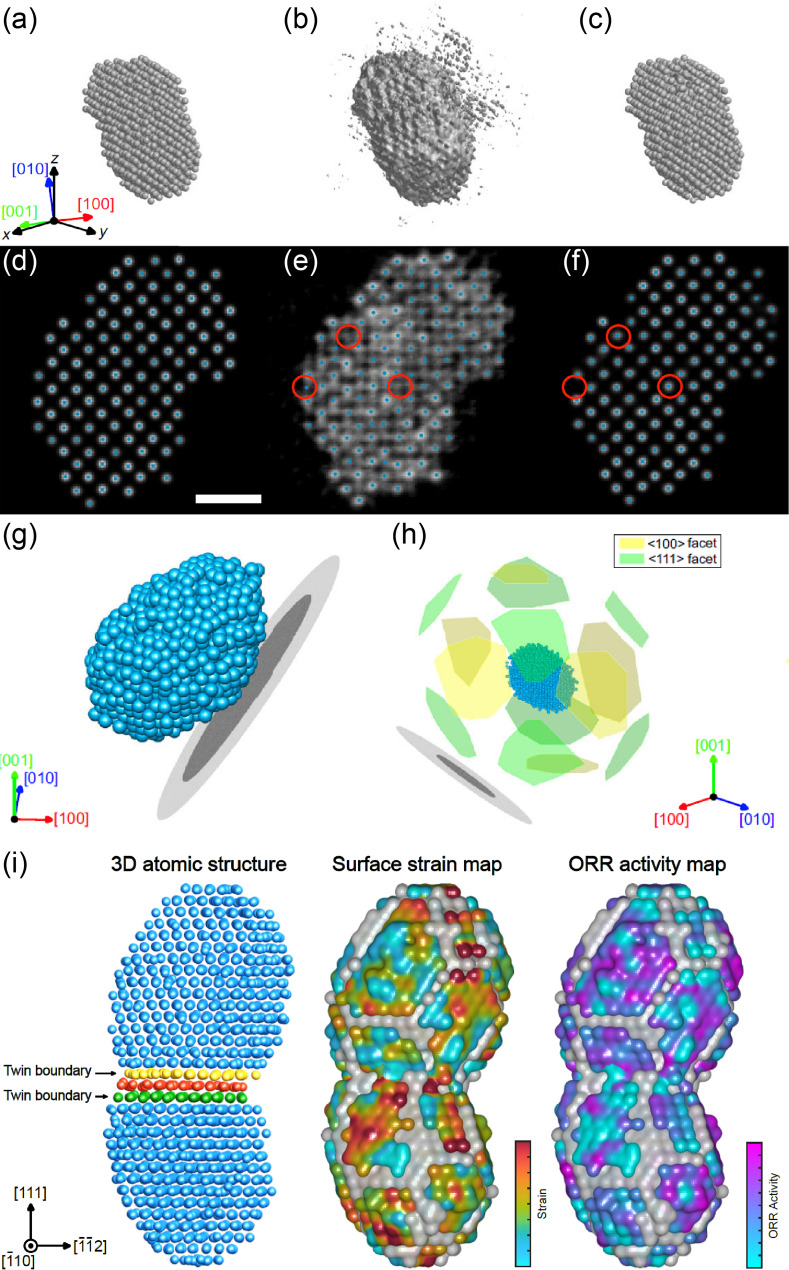
Determination of Pt nanoparticle surface and interface structures via deep learning augmentation. (**a**-**c**) 3D iso-surfaces plotted with 10% iso-surface values (10% of the highest intensity), representing ground truth (**a**), linear tomogram before (**b**) and after the augmentation (**c**) from simulation of AET process for a Pt nanoparticle. Note that the z direction is the missing wedge direction. (**d**-**f**) 2-Å-thick slices perpendicular to [001] direction, obtained from the 3D tomograms near the center region. Ground truth (**d**), linear tomogram before (**e**) and after the augmentation (**f**). The grayscale background represents the reconstructed intensity, and blue dots represent the positions of traced atoms. Red circles denote misidentified atoms before the augmentation, which become correctly traced after the augmentation. Scale bar, 1 nm. (**g**) Experimentally determined 3D atomic structures of a Pt nanoparticle obtained via AET with neural network-based augmentation. The SiN substrate appears as black and gray disks. (**h**) Identified surface facets of the Pt nanoparticle, showing both < 100 > and < 111 > facets. Adapted from Reference [108]. (**i**) 3D structure of a Pt nanodumbbell revealing a twin boundary at the interface. Surface strain, measured at atomic resolution, was directly correlated with catalytic activity through DFT calculations. Adapted from Reference [60] with permission, © 2022 American Chemical Society

The application of 3D U-Net-based augmentation has enabled reliable determination of three-dimensional surface atomic structures of platinum nanoparticles at single-atom resolution. In the first demonstration, nearly 1,500 atoms (including low-coordination surface atoms) were precisely located and identified within a single Pt nanoparticle [108] (Fig. 3a-h). The neural network augmentation significantly improved both atom detectability and positional accuracy (Fig. 3a-f): the fraction of correctly identified atoms increased from approximately 96.5 to 98.8%, and the root-mean-square deviation in atomic coordinates decreased from 26.1 pm to 15.1 pm. To validate the reliability of the determined atomic model, R-factor was computed by comparing the experimental tilt series to calculated projections derived from the reconstructed atomic structures. The R-factor improved from 19.2 to 17.4%. The lower R-factor for the deep learning-augmented tomogram indicates greater consistency with the experimental data, affirming that the reconstruction is more faithful to the true structure. Importantly, many surface atoms that were unidentifiable in the uncorrected tomogram due to signal smearing or intensity loss caused by the missing wedge became clearly resolvable after deep learning enhancement. These results allowed for detailed analysis of surface strain fields, revealing facet-dependent strain states: the {100} and {111} surfaces contributed unequally to the overall anisotropic strain distribution, with localized compressive strain identified at the particle-support interface (Fig. 3g-h).

Beyond isolated nanoparticles, neural network-assisted AET also enabled the direct visualization of the interface between coalescing Pt particles. More specifically, a dumbbell-shaped Pt nanoparticle formed by the coalescence of two clusters was reconstructed in full 3D, revealing a distinct double twin boundary at the junction and significant atomic disorder throughout the structure [60] (Fig. 3i). Using the atomic coordinates, a complete three-dimensional strain tensor was derived, uncovering strong tensile strain localized at the protruded {100} region. This experimental strain map was then used as direct input for density functional theory calculations, linking the observed strain to enhanced oxygen reduction reaction activity on the strained facet. These studies underscore the power of neural network-assisted AET in uncovering subtle structural features such as twin boundaries, strain anisotropy, and coalescence-induced disorder, while also establishing quantitative links between structure and catalytic functionality.

It is important to note, however, that the reported surface atomic structures should not be interpreted as perfectly accurate representations of static configurations. At room temperature, surface atoms can undergo spontaneous diffusion at 10^− 4^–10^− 7^ s timescale [140] even without electron beam exposure, and the high electron dose (typically on the order of 10^5^ e Å^−2^) used during AET acquisition may further perturb the surface. These limitations may introduce artifacts into the reconstructed structures. Nonetheless, the obtained surface configurations represent the most detailed experimental insight currently available, providing unparalleled information on the structural behavior of nanomaterial surfaces at atomic resolution.

### Improving AET via image inpainting

**Fig. 4:**
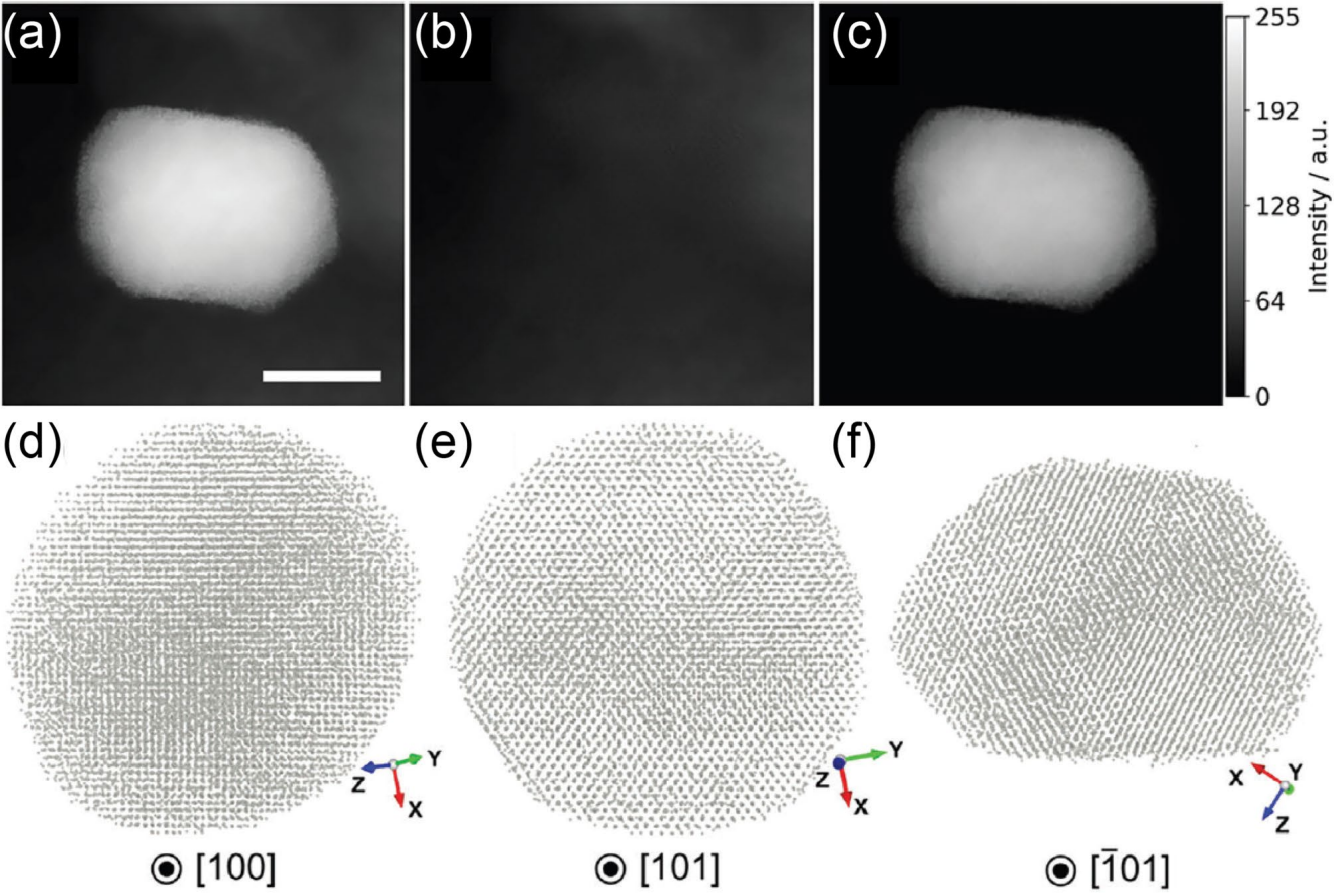
Enhancing AET through image inpainting. (**a**) A representative denoised tilt-series image of a Pd nanoparticle supported on an Al_2_O_3_ substrate. (**b**) Al_2_O_3_ background extracted from (**a**) using a CNN-based image inpainting method. (**c**) Isolated Pd nanoparticle after background removal. Scale bar, 5 nm. (**d**-**f**) Reconstructed 3D atomic structure of the Pd nanoparticle viewed along (**d**) [100], (**e**) [101], and (**f**) [101] directions. The green and blue arrows indicate the tilt axis (y-axis) and the electron beam direction (z-axis), respectively. Adapted from Reference [135] with permission, © 2023 Wiley-VCH GmbH.

In a recent development, Iwai et al. introduced a CNN-based image inpainting strategy to address challenges in imaging supported metal nanoparticles via AET [135]. Their approach focused on isolating the nanoparticle signal from the complex background contributions of the support material in electron tomography tilt series. The inpainting model effectively predicted and removed the support signal, enabling clearer visualization of the nanoparticle itself (Fig. 4a-c). Using the inpainted dataset, the team reconstructed an 11 nm palladium nanoparticle in 3D, revealing a deformed cuboctahedron structure with high-index facets (Fig. 4d-f). These morphological characteristics are associated with increased catalytic activity, particularly in methane combustion. Furthermore, atom-level mapping of local Pd-Pd bond distances and their variations enabled visualization of interfacial strain and atomic disorder at the Pd/Al_2_O_3_ boundary. This study demonstrates the utility of deep learning in preprocessing tilt series data to isolate relevant structures from noisy or overlapping signals, thereby expanding the applicability of AET to supported systems. The ability to accurately reconstruct and analyze nanoparticles in realistic environments opens new pathways for rational catalyst design and performance optimization.

### Summary and outlook

Recent advances in deep learning have significantly expanded the capabilities of AET, enabling reliable three-dimensional imaging of materials with single-atom precision, even under challenging conditions such as limited tilt ranges, strong background interference, and complex surface structures. By integrating convolutional neural networks into the AET reconstruction pipeline, researchers have developed powerful tools for recovering missing information, suppressing reconstruction artifacts, and refining atomic-resolution data.

Tomogram augmentation with neural networks, including U-Net and transformer-based models, has substantially improved reconstruction accuracy and atomic site resolution. By incorporating physical priors such as atomicity, these methods generalize across diverse structural types and have enabled precise mapping of facet, strain, and distortions in nanoparticles. When combined with theoretical modeling, they support quantitative analysis of catalytic activity at the atomic scale. In parallel, CNN-based inpainting techniques have proven effective in isolating nanoparticle signals from complex backgrounds, facilitating 3D analysis of supported catalysts. Collectively, these advances are transforming AET into a versatile, data-driven platform for atomic-scale materials characterization.

Looking ahead, further integration of deep learning with advanced AET methodologies is expected to unlock new frontiers. Conventional AET techniques based on annular dark-field measurements remain limited to relatively heavy elements due to their stronger electron scattering signals. However, many technologically relevant nanomaterials contain low-Z elements (such as oxygen, carbon, and nitrogen) alongside heavier atoms. In particular, for oxide systems, understanding their emergent properties—especially those arising at the nanoscale or at heterointerfaces—requires accurate 3D imaging of light elements such as oxygen [101, 141–143].

In this context, four-dimensional scanning transmission electron microscopy (4D-STEM) [144, 145], enabled by high-speed pixelated detectors, offers new opportunities by fully exploiting the information carried by scattered electrons in the diffraction plane. Recent experimental and computational developments suggest that 4D-STEM-based ptychography [146–152] and tomography [153–159] can resolve atomic positions of both light and heavy elements with high accuracy. Furthermore, newly developed 4D-STEM multislice tomography methods enable out-of-focus imaging, overcoming depth-of-focus limitations and allowing analysis of larger sample volumes. Similarly, multislice tomography using multiple tilted high-resolution TEM focal series offers comparable benefits. It achieves high-accuracy atomic resolution for both light and heavy elements while mitigating depth-of-focus constraints, making it especially suitable for beam-sensitive materials, such as biological samples [153, 160, 161]. When combined with neural network-based reconstruction, these developments promise full 3D atomic mapping (including light elements) across a wide range of material systems.

Another important direction is the role of neural networks in improving the interpretability and throughput of atomic-resolution tomograms. These approaches not only enhance image quality but also streamline the extraction of structural information, potentially enabling higher-throughput AET workflows. This will make it more feasible to conduct repeated measurements of structural changes under external stimuli, such as thermal annealing, mechanical stress, or electromagnetic fields, thereby facilitating studies of structural dynamics in real time [111, 162].

Unsupervised neural networks for denoising low-dose TEM images are also under active development, demonstrating promising results in atomic-resolution tomography under in situ conditions, for example, in liquid-phase TEM where reconstructions are based on the random motion of nanomaterials [110, 112, 131]. Additionally, deep learning may contribute to uncertainty quantification in atomic models, chemical species classification, and automated interpretation of structure-function relationships.

Despite these promising developments, several challenges remain. Surface atom mobility during acquisition, beam-induced artifacts, and the absence of universally validated ground truth datasets continue to limit the reliability of reconstructed structures [163–165]. Overcoming these obstacles will require ongoing advancements in both algorithm development and experimental methodologies.

Nevertheless, the convergence of deep learning and AET is already reshaping our ability to observe and understand matter at the atomic scale. Continued progress in model interpretability, generalization, and physics-informed training [166–170] will further enhance the reliability, robustness, and accessibility of AET—ultimately making high-precision 3D imaging a routine tool for nanoscience and materials research.

## Electronic supplementary material

Below is the link to the electronic supplementary material.

Supplementary Material 1

## Data Availability

Data sharing is not applicable to this article as no datasets were generated or analyzed.

## Abbreviations

3D: Three-dimensional
AET: Atomic electron tomography
2D: Two-dimensional
TEM: Transmission electron microscopy
CNN: Convolutional neural network
GAN: Generative adversarial network
IRDM: Information recovery and de-artifact model
EC-UNETR: Ensemble cross U-Net transformer model
4D-STEM: Four-dimensional scanning transmission electron microscopy
