# Supplementary material for: Advancing atomic electron tomography with neural networks
Source: Appl Microsc. 2025 Jun 19;55:7. doi: 10.1186/s42649-025-00113-7 (PMC12177128; doi:10.1186/s42649-025-00113-7)
Supplement: Supplementary file 1 — Supplementary Material 1 [file 42649_2025_113_MOESM1_ESM.pdf]

First of all, we thank the reviewers for taking their time and effort to provide a very constructive comments, which has been extremely helpful in making further improvements to the manuscript.

Please find below our response to the reviewer's comment on our manuscript "*Advancing atomic electron tomography with neural networks*". We tried our best to carefully address the suggestion and trust that the manuscript is now acceptable for publication in *Applied Microscopy*.

Response to reviewer's comment

Reviewer #1 (Remarks to the Author):

1. *This review article is very well written and provides a timely and comprehensive overview of recent developments in neural network-assisted atomic electron tomography (AET). The authors clearly articulate the technical challenges of conventional AET and effectively explain how deep learning approaches, such as convolutional neural networks and transformer-based models, have advanced 3D atomic reconstruction and analysis. Notably, the review includes well-organized case studies and highlights future prospects, including the extension to light-element imaging and dynamic structural studies. Overall, this manuscript offers valuable insights into the current state and future directions of data-driven AET, and I recommend it for publication without any revision.*

   Response: We thank the reviewer for appreciating our efforts in assembling this review article. We hope it will serve as a valuable resource for microscopists working in the relevant fields.

Reviewer #2 (Remarks to the Author):

1. *This review manuscript addresses a highly relevant and rapidly evolving topic at the intersection of electron microscopy and machine learning. The authors present a comprehensive overview of neural network applications in atomic electron tomography (AET), with particular emphasis on addressing the persistent "missing wedge" problem. The manuscript is generally well-written and covers important recent developments in the field. The manuscript excellently captures the current state of neural network integration in AET. The authors have identified a critical gap in the literature and provide a much-needed synthesis of this rapidly evolving field. The timing is particularly appropriate given the recent surge in AI applications across microscopy. The "missing wedge" problem is clearly articulated, and its impact on surface atomic structure determination is well-explained. The authors effectively communicate why this limitation is particularly critical for catalysis and surface science applications, making the motivation for neural network approaches compelling. This review will serve as an excellent resource for the electron microscopy and materials characterization communities, being well-suited for Applied Microscopy.*
   As a minor comment, some sentences seem quite long and could be broken down for better readability. In addition, please, define technical terms consistently (e.g., "atomicity principle" mentioned but not fully explained).

   Response: We thank the reviewer for appreciating our efforts in putting together this review article. As suggested, we have clarified technical terms, including the "atomicity principle," and revised long sentences into simpler expressions to enhance readability for the journal's audience. We hope this article will serve as a helpful resource for microscopists working in the relevant field.
